# Bioinformatics analysis of gene expression profile of serous ovarian carcinomas to screen key genes and pathways

**DOI:** 10.1186/s13048-020-00680-1

**Published:** 2020-07-21

**Authors:** Hongjun Fei, Songchang Chen, Chenming Xu

**Affiliations:** Department of Reproductive Genetics, International Peace Maternity and Child Health Hospital, Shanghai Key Laboratory of Embryo Original Diseases, Shanghai Municipal Key Clinical Specialty, Shanghai Jiao Tong University School of Medicine, No.910, Hengshan Road, Shanghai, 200030 People’s Republic of China

**Keywords:** Serous ovarian carcinomas, Differentially expressed genes, Tumor biomarkers, Function analysis, *KIF11*, *CDC20*

## Abstract

**Background:**

Serous ovarian carcinomas (SCA) are the most common and most aggressive ovarian carcinoma subtype which etiology remains unclear. To investigate the prospective role of mRNAs in the tumorigenesis and progression of SCA, the aberrantly expressed mRNAs were calculated based on the NCBI-GEO RNA-seq data.

**Results:**

Of 21,755 genes with 89 SCA and SBOT cases from 3 independent laboratories, 59 mRNAs were identified as differentially expressed genes (DEGs) (|log_2_Fold Change| > 1.585, also |FoldChange| > 3 and adjusted *P* < 0.05) by DESeq R. There were 26 up-regulated DEGs and 33 down-regulated DEGs screened. The hierarchical clustering analysis, functional analysis and pathway enrichment analysis were performed on all DEGs and found that Polo-like kinase (PLK) signaling events are important. PPI network constructed with different filtration conditions screened out 4 common hub genes (*KIF11*, *CDC20*, *PBK* and *TOP2A*). Mutual exclusivity or co-occurrence analysis of 4 hub genes identified a tendency towards co-occurrence between *KIF11* and *CDC20* or *TOP2A* in SCA (*p* < 0.05). To analyze further the potential role of *KIF11* in SCA, the co-expression profiles of *KIF11* in SCA were identified and we found that *CDC20* co-expressed with *KIF11* also is DEG that we screened out before. To verify our previous results in this paper, we assessed the expression levels of 4 hub DEGs (all up-regulated) and 4 down-regulated DEGs in Oncomine database. And the results were consistent with previous conclusions obtained from GEO series. The survival curves showed that *KIF11*, *CDC20* and *TOP2A* expression are significantly related to prognosis of SCA patients.

**Conclusions:**

From all the above results, we speculate that *KIF11*, *CDC20* and *TOP2A* played an important role in SCA.

## Introduction

Ovarian cancer represents the most lethal neoplasm of the female genital tract. It is the fifth most frequent cause of cancer death in women in the United States in 2019, and 5-year survival rates are less than 30% of all the women diagnosed with ovarian cancer [1]. According to the WHO statistics in 2018, each year an estimated total of 295,400 cases of ovarian cancer will be diagnosed and 184,800 patients with ovarian cancer will die from their disease over the world [2–4]. Ovarian carcinoma comprises various histologic subtypes based on the cell of origin, among the different histologic subtypes, epithelial ovarian cancer accounts for 90% of ovarian carcinoma and the serous type accounts for 75 to 80% of epithelial ovarian carcinomas [5]. So, serous ovarian carcinomas (SCA) is the most common subtype [6, 7].

SCA is mainly high grade and low-grade serous carcinoma (LGSC) represents less than 10% of all cases of SCA [8–10]. It characterized by involvement of both ovaries, aggressive behavior, late stage at diagnosis, and low survival [9]. As most common and most aggressive subtype [11, 12], yet its etiology remains unclear.

Borderline ovarian tumors (BOT) are neoplasms of epithelial origin characterized by up-regulated cellular proliferation and the presence of slight nuclear atypia but without destructive stromal invasion which also described as atypical proliferative tumors or tumors of low malignant potential (LMP) [13]. Approximately 70% BOT are serous type [14–16]. BOT differ from ovarian carcinoma by absence of stromal invasion, thus prognosis is excellent for BOT with 5- and 10-year survival of 99 and 97%, 98 and 90%, and 96 and 88% for stages I, II and III tumors, respectively [17]. There is a complete lack of biomarkers and screening methods for accurate early-stage detection of ovarian cancer. Screening may be particularly problematic for SCA [18]. Toward that end, in this study, we collected microarray expression profiles of SCA and compared it with the less malignant epithelial ovarian cancer type, serous borderline ovarian tumors (SBOT). Though our results of data analysis, we want to shed light on finding potential diagnosis and therapeutic targets of SCA.

Microarray technology is a powerful high-throughput platform for biological exploration. Gene expression profiling of cancers represents the largest research category using microarrays and appears to be the most robust approach for molecular characterization of cancers [19]. It has been widely used to determine the possible genetic or epigenetic alternations and identify biomarkers in various disorders [20, 21], and a great deal of cores slice data have been produced, most of the data was deposited and stored in public databases. Integrating and re-analyzing these data can provide valuable clues for new research. Although there has been some work focused on searching critical gene sets in SCA using gene expression data, individual investigations are always limited or inconsistent due to tissue or sample heterogeneity in independent studies or the results were generated from a single cohort study. With our study, by means of integrated bioinformatics analysis of available expression profiling microarray data from different laboratories, statistical power increased and prediction is more accurate, moreover, bias of individual studies can be overcoming. So, it is possible to come up with more reliable and precise screening results via overlapping relevant data sets.

In the present study, we have downloaded 3 original microarray datasets GSE36668 (4 SBOT samples and 4 SCA samples), GSE27651 (8 SBOT samples and 35 SCA samples) and GSE12471 (13 SBOT samples and 25 SCA samples) from NCBI-Gene Expression Omnibus database (NCBI-GEO) (Available online: https://www.ncbi.nlm.nih.gov/geo), from which there were total of 25 SBOT cases and 64 SCA tissues available. Subsequently, the differentially expressed genes (DEGs) were screened using R language and 59 DEGs were filtered out from 21,755 genes based on 3 independent datasets which contained 89 ovarian carcinoma cases. To better clarify the pathological mechanisms of SCA, we performed cluster analysis, functional analysis and biological pathway and process enrichment analysis for 59 screened DEGs. To determine hub genes with significant expression difference between SCA and SBOT, we constructed protein-protein interaction (PPI) network for 248 DEGs screened with the threshold of |log_2_FoldChange| > 1.0 and 59 DEGs screened with the threshold of |log_2_FoldChange| > 1.585 respectively. Then, 13 hub genes and 6 hub genes were screened out based on different thresholds, and the intersection of two sets were performed. Therefore, we obtained 4 most important hub genes: *KIF11*, *CDC20*, *PBK* and *TOP2A*. To verify our screening results, the expression signatures of the 4 hub DEGs in clinical cancer tissue were assessed by several databases. Their expressions in normal ovary and SCA tissues were analyzed in oncomine database. The co-expression analysis of the 4 hub DEGs was conducted by cBioportal reveals the co-occurrence or mutual exclusivity relationship and provided the information for the possible underlying mechanism. The survival of ovarian cancer and SCA patients with high or low DEGs expressions were identified with KM plotter database. All in all, we hope to gain further insight of SCA at molecular level and explored the potential candidate biomarkers for diagnosis, prognosis, and drug targets.

## Materials and methods

### Microarray data selection

In the current study, the gene expression profiling data sets (ID: GSE36668, GSE27651, GSE12471) were obtained from Gene Expression Omnibus database of the National Center for Biotechnology Information (NCBI). We used “serous ovarian cancer”, “*Homo sapiens* [organism]” and “expression profiling by array [dataset type]” as the keywords in the GEO database. There were 167 results under this search condition. The microarray datasets were selected according to the following rules: the samples must contain human SBOT and SCA tissues; the patients did not receive special treatment, including radiotherapy and chemotherapy; and dataset tested genes cannot less than 8000. Under these conditions, we obtained 3 datasets to performing further analyze although there were 5 datasets contain SBOT and SCA tissues (GSE3208 and GSE17308 were excluded because different microarray type induced very difference expression data). In other words, data we used were extracted from the original studies by 3 independent researchers. The following information was extracted from each identified study: GEO accession number, sample type, platform, number of SBOT and SCA tissues, and gene expression datas. The information of the selected GEO series was listed in Table 1. We download the raw data of 89 specimens from 3 independent GEO series. Totally 25 SBOT and 64 SCA specimens were enrolled in GSE36668, GSE27651 and GSE12471 (platform: GPL570 Affymetrix Human Genome U133 Plus 2.0 Array and GPL201 Affymetrix Human HG-Focus Target Array). The process of data filing is showed in Supplementary Figure 1.

**Table 1:** Characteristic of included microarray data

Expression profiling array (SBOT & SCA)	Platforms	GEO accession	Samples
Genome	GPL570	GSE36668	4 SBOT; 4 SCA
		GSE27651	8 SBOT; 35 SCA
	GPL201	GSE12471	13 SBOT; 25 SCA

### Data preprocessing before difference analysis

We utilized the Robust Multi-array average algorithm of the Affy package in R language to convert the raw data to expression data. The expression levels of the probe sets were converted into gene expression levels by Bioconductor annotation function of R language according to the platform annotation files. Expression values of multiple probes for a given gene were averaged. With this, we obtained 3 tables containing expression value of tested genes based on 3 GEO series. Then, the function called sameGene in R language was used to merge the gene expression data of 89 patients from datasets of GSE36668, GSE27651 and GSE12471 into one output table according to the gene symbol. Then the datasets of the output table were assigned into 2 groups: SBOT group and SCA group. Batch normalization was conducted on all expression profiling data using ComBat algorithm in Surrogate Variable Analysis package of R language. The normalization can eliminate the systematic variations among different studies.

### Differentially expressed genes (DEGs) screening

The DEGs were selected from the normalized data of SBOT and SCA tissues using the linear models for microarray data (Limma) package in Bioconductor (http://www.bioconductor.org/packages/release/bioc/html/limma.html). Results with |log_2_FoldChange| (|log_2_FC|) > 1.585, also known as |FoldChange| > 3 and adjusted *P*-value < 0.05 were considered significant.

Volcano plot, representing the distribution of the fold change and *p*-value of all genes was drawn. Heat map of expression hierarchical clustering analysis for top 50 genes was performed to investigate probable discrepancies between SBOT and SCA tissues.

### Functional and pathway enrichment analysis for all DEGs

To gain biological sights of involved DEGs, we did functional enrichment analysis with FunRich. The FunRich software is a standalone functional enrichment and network analysis tool. It was utilized to perform Cellular component, functional (Molecular function and Biological process) and pathway (Biological pathway) enrichment analysis for obtained DEGs with *p* value < 0.05 as a strict cutoff.

### Protein–protein interaction (PPI) network construction and hub genes identification

The functional protein–protein interaction (PPI) analysis is essential to interpret the molecular mechanisms of key cellular activities in carcinogenesis. It is constructed on the basis of Search Tool for the Retrieval of Interacting Genes (STRING) database [22]. Our study used the database to construct PPI network of all DEGs. Interaction score of 0.4 was regarded as the cut-off criterion and the PPI was visualized.

Hub genes were selected with interaction degree > 18 in the condition of |log_2_FoldChange| > 1.585 and interaction degree > 48 in the condition of |log_2_Fold Change| > 1.0. Venn’s diagrams were used to find intersection of the two hub genes sets selected with different condition and finally there are 4 hub genes we selected were highly interconnected with other nodes.

### Genetic alteration and co-expression analysis of 4 screened hub DEGs

The cBioPortal (http://www.cbioportal.org) [23] is an open-access resource for interactive exploration of multidimensional cancer genomics data sets. We studied alterations (amplification, deep deletion, missense mutation, inframe mutation, truncating mutation, mRNA upregulation and mRNA downregulation) in *KIF11*, *CDC20*, *PBK* and *TOP2A* genes in Ovarian Serous Cystadenocarcinoma (TCGA, provisional) case set using cBioPortal. The cBioPortal is also used for co-occurrence or mutual exclusivity and customizable correlation analysis.

### Oncomine database analysis and Kaplan-Meier plotter analysis for DEGs

Oncomine [24, 25] is a cancer transcriptomic database and web-based discovery platform with genome-wide expression analyses of various cancers. The expression level of 4 screened hub DEGs were analyzed using Oncomine Cancer Profiling Database (https://www.oncomine.org). The expression fold change of mRNA in SCA tissues compared to normal ovary tissues were obtained and compared. Co-expression analysis in Oncomine was used to identify sets of genes with synchronous expression patterns. The co-expression profiles of *KIF11* in SCA was identified and presented as the pattern of heat map.

The Kaplan–Meier plotter is a database that can be used to assess the effect of 54,675 genes on patient survival using 10,461 cancer samples (breast, ovarian, lung and gastric cancer) [26]. For survival analyses, the prognostic value of 4 screened hub DEGs in ovarian cancer and SCA were analyzed using Kaplan-Meier Plotter (http://kmplot.com/analysis/) and tested for significance using log-rank tests [27]. The analysis was performed according to the manufacturer’s instructions.

## Results

### Normalization of gene expression data

Expression data of 21,755 genes from 89 samples (25 SBOT and 64 SCA specimens) were normalized with median method following batch normalization. The expression values of all specimens before and after normalization were showed by the top and bottom box figures in Supplementary Figure 2. Horizontal axis stands for different samples.

Vertical axis stands for gene expression value. Black horizontal line represents the median of expression value of sample, which is almost on a straight line after batch normalization, suggesting that normalized data were qualified.

### Selection of DEGs and expression hierarchical clustering analysis

We used R Limma package software to analyze which gene sets were aberrantly expressed in comparisons with the threshold of |log_2_FC| > 1.585 and *P* < 0.05. The DEGs were identified using t tests statistic algorithm. The significant genes’ lists were selected according to fold change of genes expression values.

**Fig. 1:**
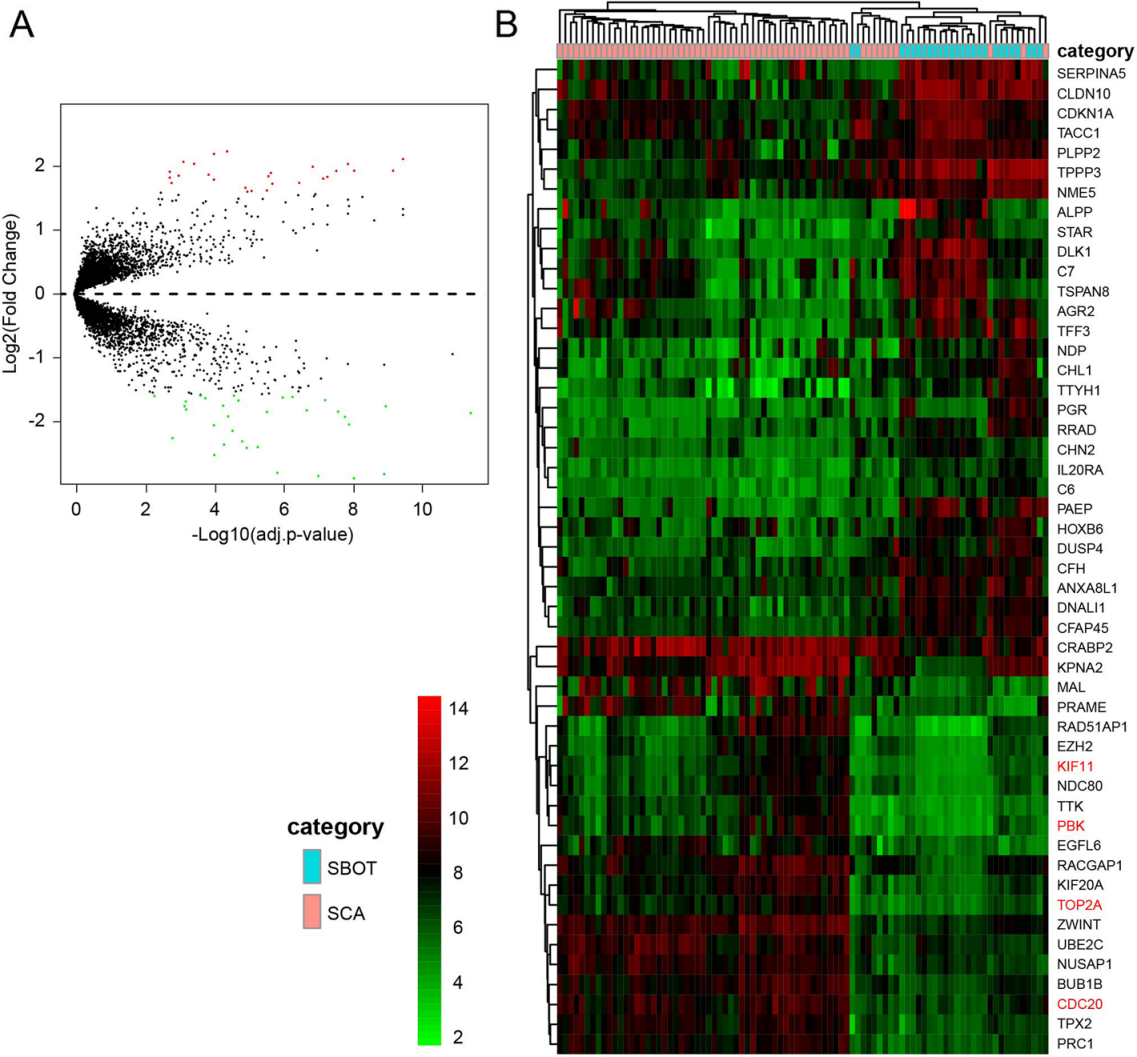
Volcano plot of the aberrantly expressed genes (**a**). The red spots represent up-regulated genes which |Log_2_FoldChange| > 1.585; The green spots represent down-regulated genes which |Log_2_FoldChange| > 1.585. Black spots show the genes with expression of |Log_2_FoldChange| < 1.585. Heat map of expression hierarchical clustering analysis for top 50 DEGs filtered from 89 specimens (**b**)

In total, 59 DEGs (26 up-regulated and 33 down-regulated) obtained based on genes expression data of 89 patients (25 SBOT and 64 SCA from 3 GEO series). We list all DEGs according to fold change of genes expression value in Table 2. The volcano plot (Fig. 1a) showed the distribution of all DEGs. Volcano plot distributions of fold change [(log_2_FoldChange] (Y-axis) and *p*-values [−log_10_ (p-value)] (X-axis).

**Table 2:** 59 DEGs, either up- or down-regulation in SCA, screened between SBOT tissues and SCA tissues from GSE36668, GSE27651 and GSE12471

Gene	Log_2_FC	*P* Value
Up-regulated genes		
PRAME	2.2157039	6.66E-07
MAL	2.1764411	2.04E-06
TPX2	2.0962153	1.89E-13
PTH2R	2.0565994	2.46E-05
RAD51AP1	2.0249715	1.06E-05
KIF20A	2.0195634	3.02E-11
CRABP2	1.9791989	5.63E-10
BUB1B	1.9182330	4.48E-13
PRC1	1.9181787	1.30E-11
CDC20	1.9142610	7.54E-11
COL11A1	1.9016324	7.99E-05
TTK	1.8800451	1.67E-08
KPNA2	1.8553682	3.19E-06
CXCL10	1.8378545	3.70E-05
EGFL6	1.8318003	2.09E-08
TOP2A	1.8184118	1.51E-10
PCP4	1.8068412	7.93E-05
ZWINT	1.7943485	2.25E-10
PBK	1.7781112	2.09E-06
UBE2C	1.7276703	1.77E-09
CENPF	1.7230898	6.80E-05
RACGAP1	1.7125167	1.49E-08
NDC80	1.6509884	1.45E-07
NUSAP1	1.6087309	2.43E-08
KIF11	1.5991830	8.70E-08
EZH2	1.5897870	1.17E-07
Down-regulated genes		
PAEP	−2.9789360	1.84E-12
CLDN10	−2.8650849	1.41E-11
DLK1	−2.8312365	3.34E-10
TPPP3	−2.7996216	1.19E-12
SERPINA5	−2.7812230	9.94E-09
ALPP	−2.5023255	1.96E-06
AGR2	−2.3902955	1.28E-07
C7	−2.3807599	4.98E-08
TSPAN8	−2.3403107	8.61E-07
TFF3	−2.2942665	1.87E-07
CRISP3	−2.2453871	6.26E-05
TTYH1	−2.1345089	4.17E-07
NDP	−2.0460981	2.07E-06
RRAD	−2.0333231	2.26E-11
CDKN1A	−1.9145302	3.86E-11
STAR	−1.9072353	5.99E-07
IL20RA	−1.8583864	4.31E-16
NME5	−1.8379724	2.39E-08
CFH	−1.8349993	6.39E-11
DNALI1	−1.8128874	9.03E-10
CHL1	−1.7992374	1.96E-05
IGFBP4	−1.7498663	2.23E-05
C6	−1.7497912	8.57E-13
PGR	−1.7410010	9.46E-07
LCN2	−1.6756923	2.00E-05
DUSP4	−1.6606638	2.58E-07
CHN2	−1.6532785	1.91E-10
HOXB6	−1.6262054	4.22E-06
ANXA8L1	−1.6123125	6.90E-09
CFAP45	−1.6037927	3.33E-09
ID1	−1.5901238	0.000292
PLPP2	−1.5878691	6.12E-06
TACC1	−1.5846984	3.68E-07

We list 26 up-regulated genes (log_2_FC > 1.585)

We list 33 down-regulated genes (log_2_FC > 1.585)

In Fig. 1b, Fold change patterns of top 50 highly DEGs were selected, analyzed and displayed in a heat map to evaluate and compare differences in gene expression between SBOT and SCA.

### Function and pathway enrichment analysis of all DEGs

**Fig. 2:**
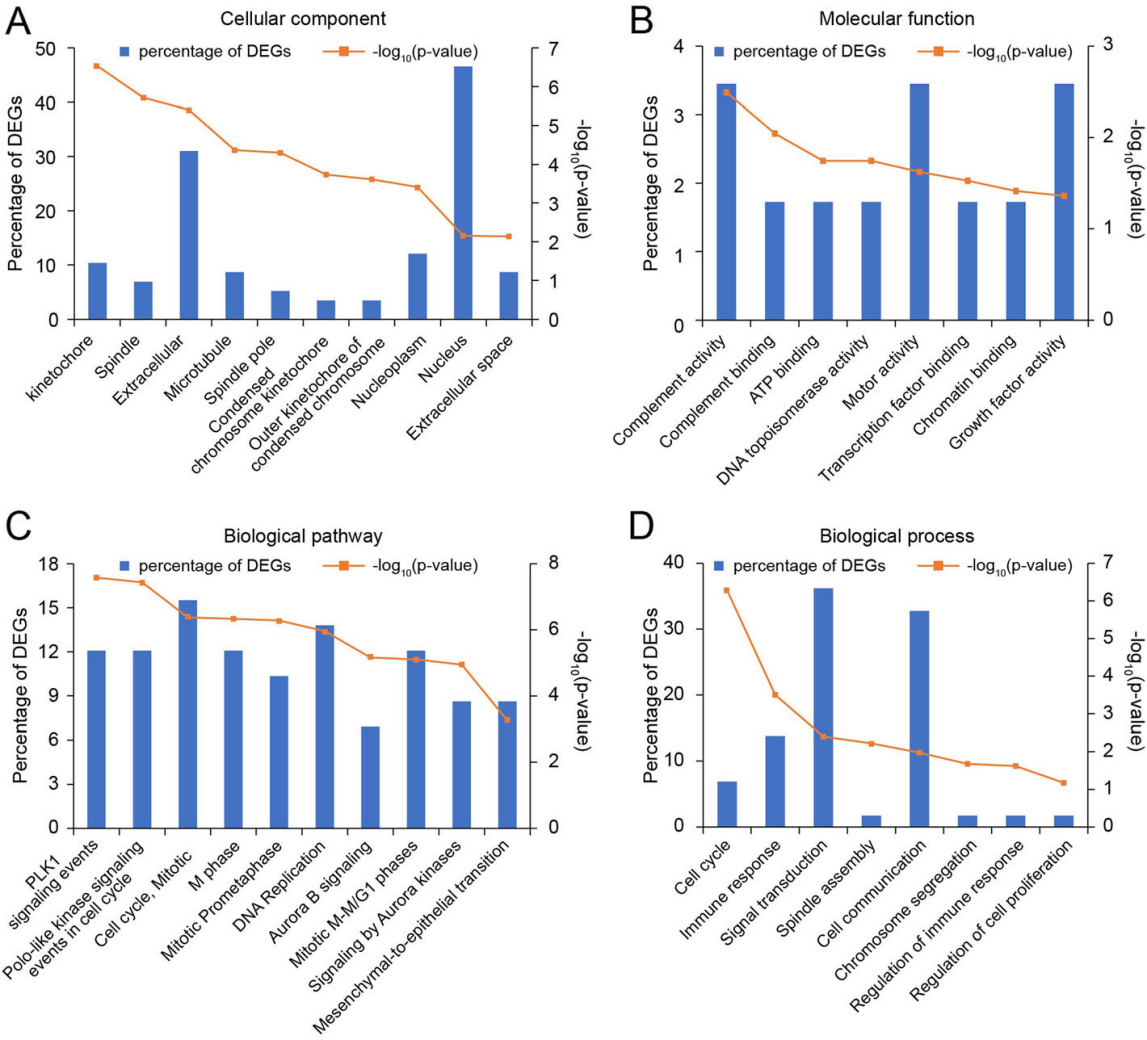
Cellular component (**a**), molecular function (**b**), significant biological pathways (**c**) and biological processes (**d**) enrichment analysis of 59 differentially expressed genes (DEGs). The Y axis represents the percentage of DEGs and -log_10_ (*p*-value), the X axis represents enriched cellular components, molecular functions, biological processes and pathways

Cellular component enrichment analysis of all DEGs described their distribution and structure (Fig. 2a). About molecular function, the DEGs significantly enriched in complement activity, complement binding, ATP binding, DNA topoisomerase activity and motor activity (Fig. 2b). To better clarify the pathological mechanisms, we performed biological pathway enrichment analysis. According to the result of pathway enrichment analysis, DEGs mainly enriched in polo-like kinase signaling (PLK1) signaling events, polo-like kinase signaling (PLK1) events in cell cycle, mitotic cell cycle and so on (Fig. 2c).

In order to further investigate the biological effects of aberrantly-expressed DEGs in SCA, biological process enrichment analysis of 59 screened DEGs was carried out. The top 8 enriched biological processes are shown in Fig. 2d. The functions in the biological process category were enriched in cell cycle, immune response, signal transduction, cell communication and so on.

### PPI network construction and hub gene selection

**Fig. 3:**
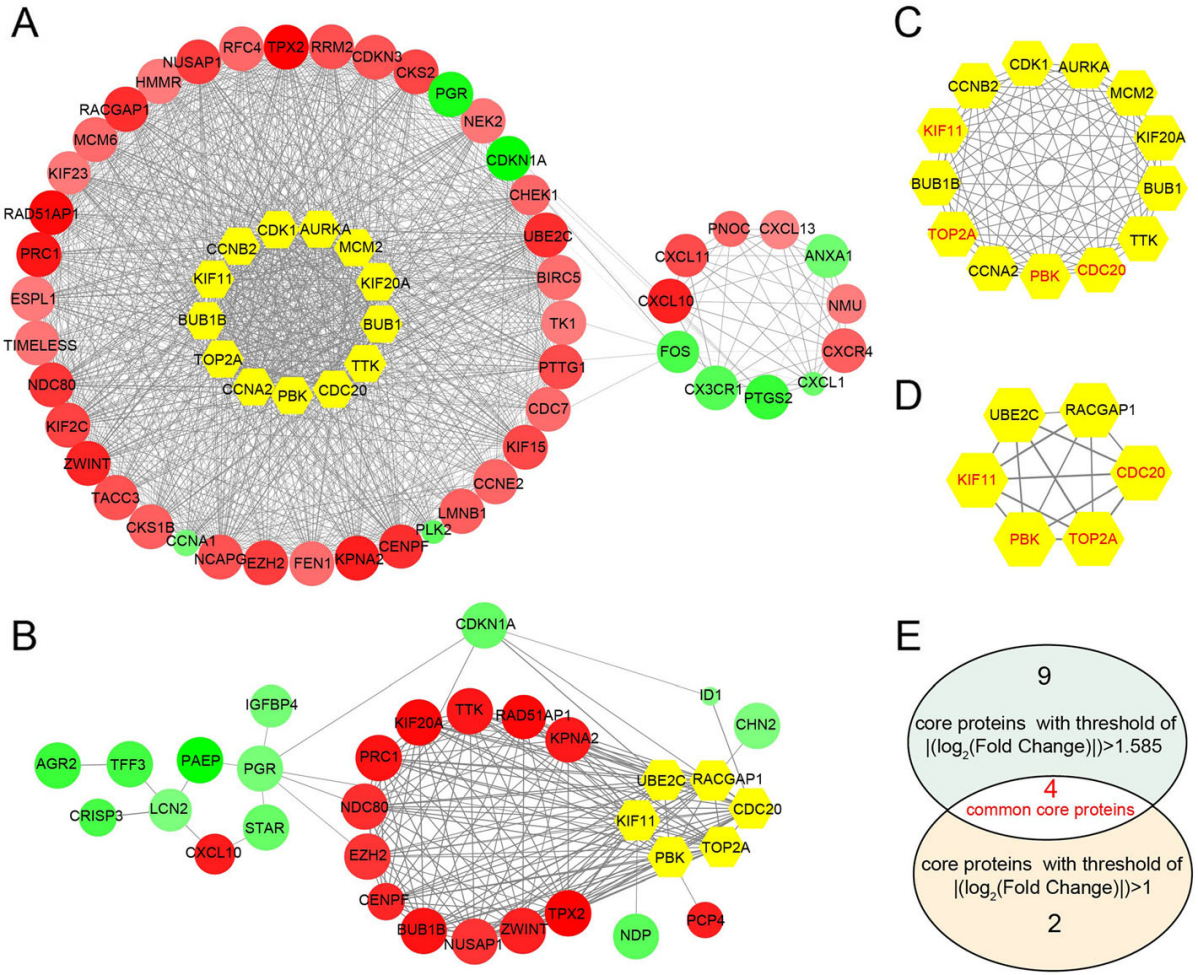
PPI network of 248 DEGs using |log_2_FoldChange| > 1.0 as screening index (**a**) and PPI network of 59 DEGs using |log_2_FoldChange| > 1.585 as screening index. The color of nodes is according to log_2_FoldChange, red nodes denotes up-regulated DEGs which log_2_FoldChange > 0 and green nodes denotes down-regulated DEGs which log_2_FoldChange < 0. The width of edge is positive correlation with combined score of protein interaction. The size of nodes is based on p-value. Yellow nodes denotes core DEGs also called hub genes. Hub genes were screened out from 248 DEGs (**c**) and from 59 DEGs (**d**) respectively. The intersection of two hub genes were obtained according to different filter criteria (**e**). Common hub DEGs were marked red

Based on the information in the STRING protein query from public databases, we made the PPI network of 248 DEGs using |log_2_FoldChange| > 1.0 as screening index (Fig. 3a), there are 13 hub genes selected with interaction degree > 48. Then, we constructed the PPI network for 59 DEGs using |log_2_FoldChange| > 1.585 as screening index (Fig. 3b), there are 6 hub genes selected with interaction degree > 18. We listed corresponding module of hub genes in Fig. 3c and d. The intersection of two hub genes sets obtained according to different filter criteria was get and showed in Fig. 3e. Top 4 hub genes were *KIF11*, *CDC20, PBK* and *TOP2A*.

### Co-expression analysis and genetic alterations of obtained hub DEGs in SCA

**Fig. 4:**
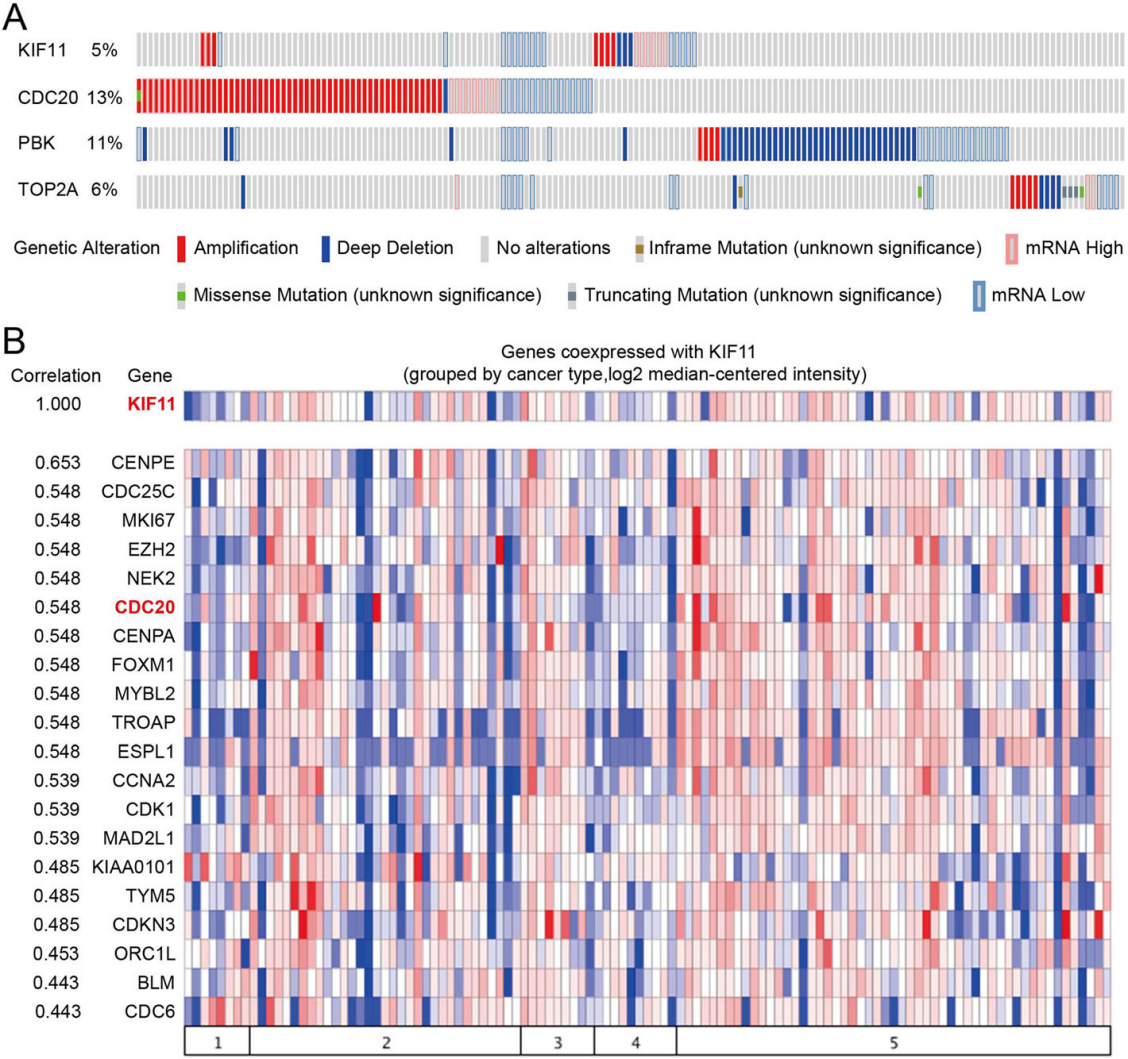
Oncoprint showing genomic alterations of hub genes in human ovarian serous cystadenocarcinoma samples based on the integrative genomic profiling in cbioportal. Genetic alterations analysis of 4 hub genes (**a**) and co-expression profiles analysis of *KIF11* (**b**)

The OncoPrint from cBioPortal is a concise and compact graphical summary of genomic alterations in multiple genes across a set of tumor samples. It summarized distinct genomic alterations including mutations, CNAs (amplifications and homozygous deletions), and changes in gene expression or protein abundance. Based on previous results of difference analysis and PPI networks, *KIF11*, *CDC20*, *PBK* and *TOP2A* were hub genes highly interconnected with other DEGs. We analyzed genomic alterations of 4 hub DEGs using cBioPortal and visualizing gene alterations across a set of SCA cases **(**Fig. 4a**)**. OncoPrints can also help identify trends such as mutual exclusivity or co-occurrence between genes. The mutual exclusivity from cBioPortal can be exploited to identify previously unknown mechanisms that contribute to oncogenesis and cancer progression, so we used cBioPortal to explore the potential relationship between 4 hub genes. As Table 3 showed, there was a tendency towards co-occurrence between *KIF11* and *CDC20* or *TOP2A* in SCA (*p* < 0.05).

**Table 3:** Co-occurrence or mutual exclusive alterations of 4 hub genes

Gene A	Gene B	*p*-value	Log odds ratio	Association
KIF11	CDC20	0.008	1.971	Tendency towards co-occurrence
KIF11	TOP2A	0.039	2.269	Tendency towards co-occurrence
CDC20	PBK	0.326	0.440	Tendency towards co-occurrence
KIF11	PBK	0.492	0.350	Tendency towards co-occurrence
PBK	TOP2A	0.559	−0.610	Tendency towards mutual exclusivity
CDC20	TOP2A	0.592	−0.229	Tendency towards mutual exclusivity

The query contains 2 gene pairs with mutually exclusive alterations (no significant), and 4 gene pairs with co-occurrent alterations (2 significant)

Log odds ratio > 0: Association towards co-occurrence

Log odds ratio < = 0: Association towards mutual exclusivity

*P*-value < 0.05: Significant association

*P*-value: Derived from Fisher Exact Test

Log odds ratio: Quantifies how strongly the presence or absence of alterations in gene A are associated with the presence or absence of alterations in gene B in the selected tumors

Co-expression analysis in Oncomine was used to identify sets of genes with synchronous expression patterns. The co-expression profiles of *KIF11* in SCA was identified and presented as the pattern of heat map. We identified the co-expression profiles for *KIF11* with a strong cluster of top 10% genes across a panel of 86 SCA tissues. Moreover, we found that *CDC20* co-expressed with *KIF11,* and they were also DEGs that screened out from SCA based on our previous results **(**Fig. 4b**)**.

### Validation of the expression of obtained hub DEGs in Oncomine database

**Fig. 5:**
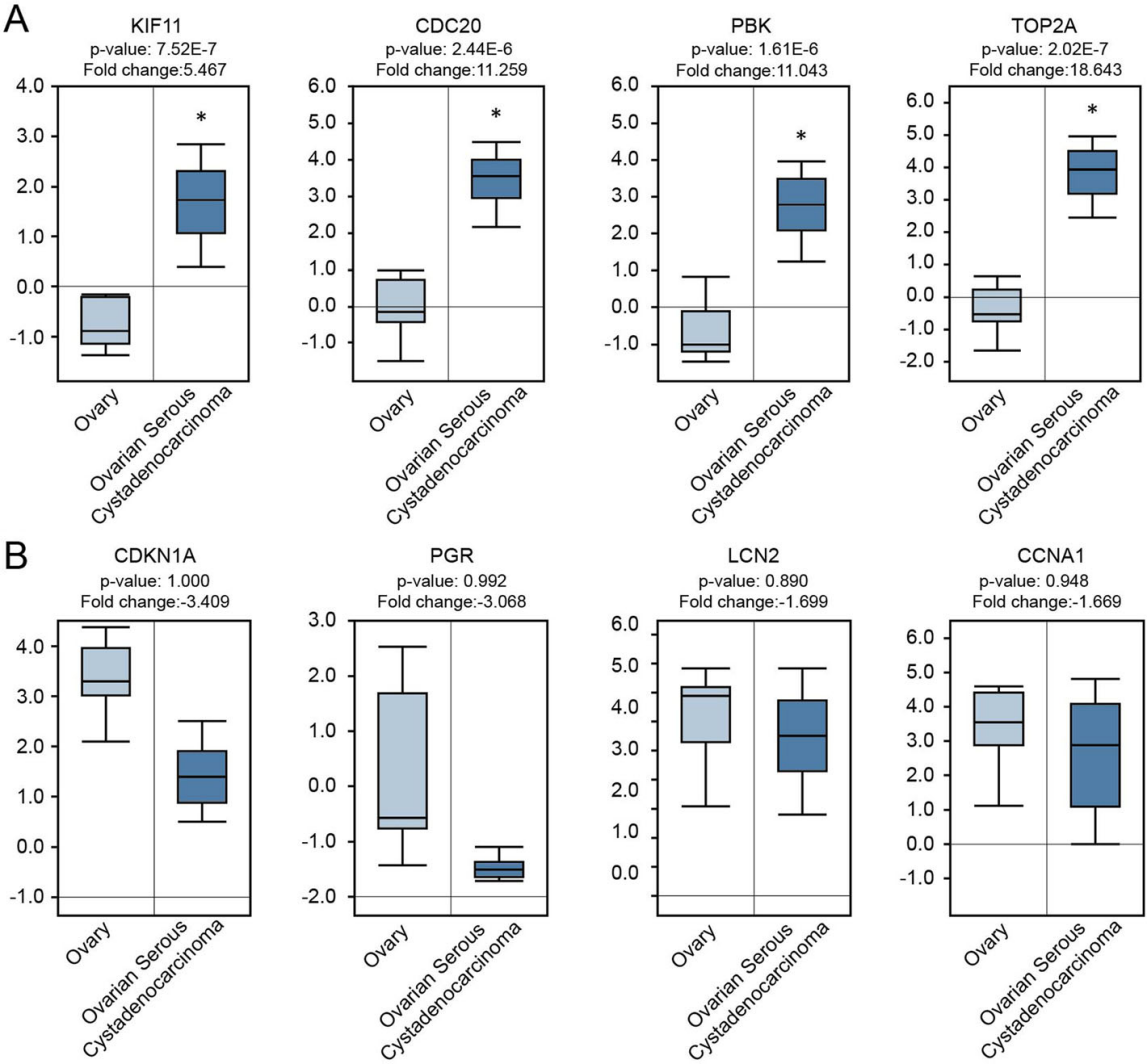
Compare expression of 4 hub genes (all up-regulated) and 4 down-regulated DEGs between SBOT and SCA tissues in Oncomine database. Box plots derived from gene expression data in Oncomine database comparing expression of the hub DEGs (**a**) and down-regulated DEGs (**b**) in SBOT (light blue columns) and SCA tissues (dark blue columns). The X axis indicates tissue types. The Y axis represents normalized expression of mRNAs

To further elucidate whether the expression of the DEGs were correlated with our analysis result on the basis of GEO data, a clinical study was performed in the light of previous results in cancer microarray database of Oncomine. The expression of 4 hub DEGs were verified in Fig. 5a. Very Coincidentally, the hub genes we screened out were all up-regulated DEGs in SCA. So, we selected 4 down-regulated DEGs which have most obvious expression changes according to heat map clustering analysis result for further analysis. The top 4 aberrantly decreased expressed DEGs were *CDKN1A*, *PGR*, *LCN2* and *CCNA1* (Fig. 5b). The results showed that the expression of hub genes and selected DEGs were consistent with our previous studies in accordance with data from GEO series. The differences had statistical significance in hub genes (*p* < 0.001), but not statistically significant in selected down-regulated DEGs although the expression of DEGs had trend of down-regulated in SCA.

### Survival analysis for obtained hub DEGs with Kaplan–Meier plotter

**Fig. 6:**
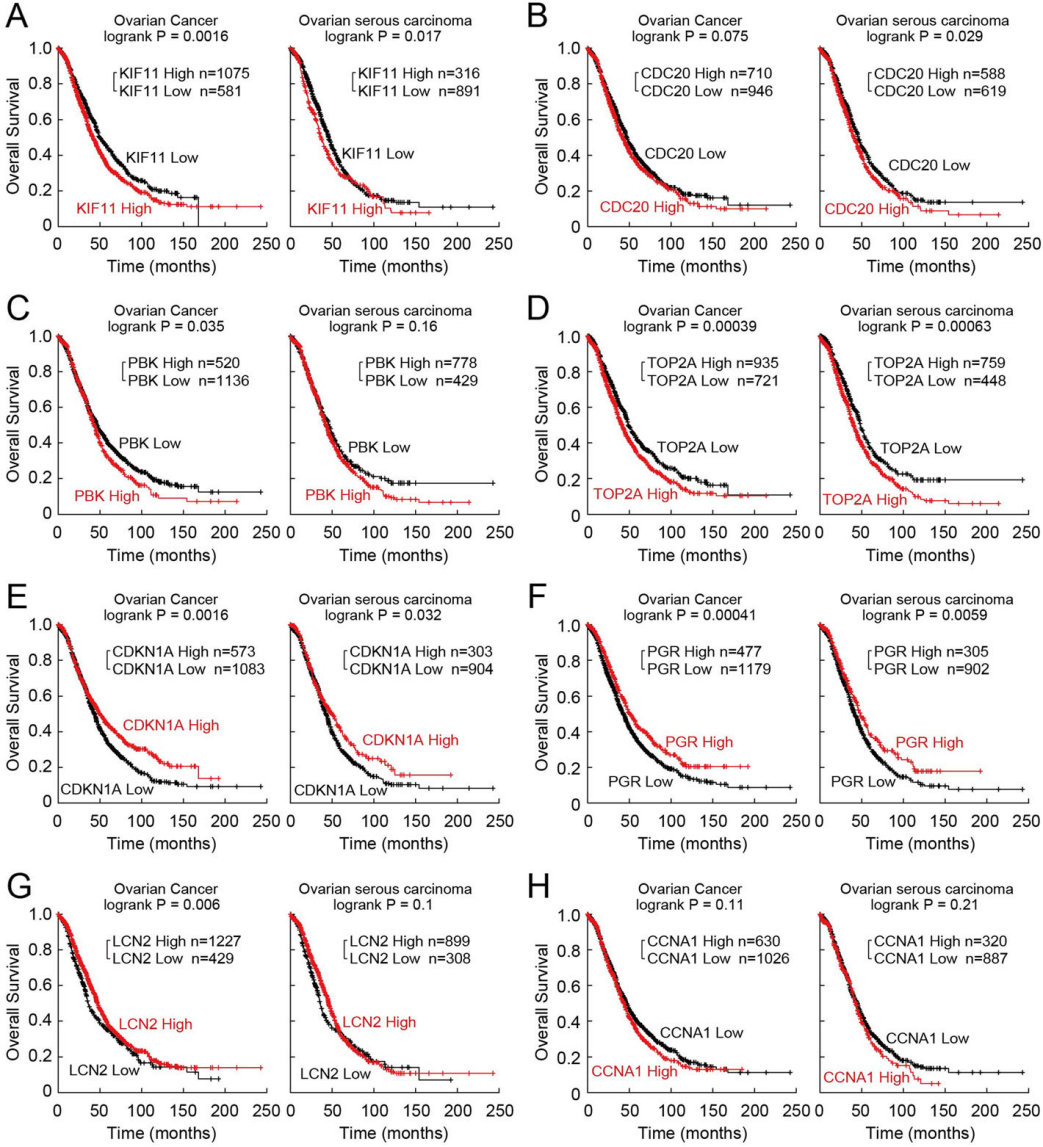
Prognostic value of 4 hub genes (**a**, **b**, **c**, **d**) and 4 down-regulated DEGs (**e**, **f**, **g**, **h**) in ovarian cancer and SCA. Data were obtained from the Kaplan–Meier plotter database. The *P* value was calculated by a log-rank test. The version of the data collected in KM plotter was 2020.04.15 version

According to our previous bioinformatics analyses and validation, the expression of 4 hub genes was up-regulated obviously in SCA, and the expression of another DEGs had trend of down-regulated in SCA. To explore the association of 4 hub genes (*KIF11*, *CDC20, PBK*, *TOP2A*) and 4 down-regulated DEGs (*CDKN1A*, *PGR*, *LCN2* and *CCNA1*) expression with the prognosis of SCA, the survival curves were drawn using Kaplan-Meier plotter database. As show in Fig. 6, the high expression of *KIF11, CDC20, PBK, TOP2A* were associated with worse prognosis and the low expression of *CDKN1A*, *PGR*, *LCN2* and *CCNA1* were associated with worse prognosis. However, in SCA patients, the effect of *PBK*, *LCN2* and *CCNA1* expression on cancer progression was not statistically significant (*p* > 0.05). But on the basis of the figures, the 3 *DEGs* showed diverse survival times, statistical nonsense may on account of insufficient samples.

## Discussion

Worldwide, approximately 295,400 women are diagnosed with ovarian cancer each year, and 184,800 are expected to succumb to the disease in 2018 [2, 3]. The case-to-fatality ratio of ovarian cancer is nearly three times that of breast cancer, and makes it the most deadly gynecologic malignancy in developed countries [28]. The vast majority of ovarian cancers are epithelial ovarian cancers which accounts for over 95% of the ovarian malignancies, and about 75% of epithelial ovarian cancers are serous type. Moreover, high-grade serous epithelial ovarian cancer is the most common subtype and accounts for up to 70% of all ovarian cancer cases [29], it is associated with worse outcomes including poor prognosis and a high risk of distant recurrence and death [30]. Hence, ovarian carcinomas of the serous histological type are an attractive target for early detection as they are rarely detected before they reach an advanced stage, when they are highly lethal. We know surprisingly little about the target for early detection of SCA. Consequently, there is an urgent need for diagnostic molecular features or biomarkers that can be associated with survival and disease recurrence in SCA.

A field which has recently contributed significantly to improved diagnostics, classification and prognostics is SCA transcriptomics microarray, a whole transcriptome high throughput sequencing and analysis technique which identifies changes in the RNA expression, is now being used to gain a more detailed understanding of the molecular mechanism of SCA [31]. Employ analysis of whole transcriptome sequencing results from different laboratories, statistical power increased and prediction is more accurate, moreover, bias of individual studies can be overcoming. In the current study, we focused on the aberrantly expressed mRNAs in SCA based on GEO RNA-seq data and the common DEGs that screened out from different researchers containing 89 samples were listed. There were 26 up-regulated DEGs and 33 down-regulated DEGs in SCA with the threshold of |log_2_FC| > 1.585 and *P* < 0.05.

Biological pathway analysis of all DEGs showed that the DEGs were mainly involved polo-like kinase (PLK) signaling events, polo-like kinase (PLK) signaling events in cell cycle, and mitotic cell cycle, biological process of all DEGs mainly include cell cycle, immune response and signal transduction. There is abundant evidence that Polo-like kinase (PLK) isoforms play an important role in a number of intracellular signal transduction pathways related to mitosis [32]. W Weichert et al had reported that PLK isoform expression is a prognostic factor in epithelial ovarian carcinoma [33]. Monika Raab et al had also demonstrated that high Polo-like kinase (PLK) 1 expression correlates with bad prognosis in epithelial ovarian cancer patients [34]. Some researchers are trying to use PLK1 inhibitors for treatment of SCA [35]. Function analysis can help us better understanding the mechanism of SCA and provide guide for SCA prevention and treatment, however, further laboratory and clinical researches are required.

PPI network of 248 DEGs using |log_2_FoldChange| > 1.0 as screening index and 59 DEGs using |log_2_FoldChange| > 1.585 as screening index helped us found 4 common hub DEGs which had most functional connections: *KIF11*, *CDC20, PBK* and *TOP2A.* OncoPrints helped us identify trends such as mutual exclusivity or co-occurrence of screened hub genes. We found that there was a tendency towards co-occurrence between *KIF11* and *CDC20* or *TOP2A* in SCA (*p* < 0.05). Then, co-expression analysis with oncomine database for *KIF11* found that *CDC20* co-expressed with *KIF11* in SCA*,* and they were also DEGs that screened out from SCA based on our previous results. Our results seem showed that *KIF11* and *CDC20* play a role in SCA. *KIF11* encodes kinesin Eg5, a motor protein required for microtubule antiparallel sliding during mitosis that has been targeted clinically. Rebecca J. Wates et al had provided new possible therapies for epithelial ovarian cancer though targeting the *KIF11/KIF15/TPX2* axis although it is still immature [36]. Some paper reported that *CDC20* overexpression is associated with development and progression of hepatocellular carcinoma [37], lung adenocarcinoma [38], and breast cancer [39]. The relationship between *CDC20* and serous epithelial ovarian cancer is still underway.

To verify our previous results in this paper, we assessed the expression levels of 4 hub DEGs and top 4 DEGs with most obvious fold changes. The expression levels of *KIF11, CDC20, PBK, TOP2A*, *CDKN1A*, *PGR*, *LCN2* and *CCNA1* were analyzed in Oncomine database, respectively. From all above results, we speculate that *KIF11*, and *CDC20* play an important role in SCA. The survival curves show that the probability of SCA progression was found to be statistically significant with high *KIF11* and *CDC20* expression as compared to that of low *KIF11* and *CDC20* expression (*p* < 0.01).

This study had several limitations. First, the survival curves of *PBK* in SCA was not statistically significant (*p* > 0.05), but on the basis of the figure, differentially expressed *PBK* have diverse survival times, statistical nonsense may on account of insufficient samples. Second, even though we performed preliminary validation of the results, more in-depth studies are needed in the future. Therefore, we hope that these results can be integrated into future experiments and facilitate further understanding of the molecular mechanisms of SCA.

Despite these limitations, we believe that this analysis represents a valuable resource and can be considered as a preliminary study for future studies of SCA. Our study provides information for researchers to identify possible candidate genes and pathways which may be involved in SCA for further studies. We gained further insight of SCA carcinogenesis at molecular level and explored the potential candidate biomarkers for diagnosis, prognosis, and drug targets.

## Conclusions

Our study utilized analysis of whole genome sequencing results from different laboratories, screened out DEGs from different sequencing platforms containing 89 samples. There were 26 up-regulated DEGs and 33 down-regulated DEGs in SCA with the threshold of |log_2_FC| > 1.585 and *P* < 0.05. Biological process analysis, biological pathway analysis, and PPI network analyses provided a set of related genes and pathways to help elucidate the molecular mechanisms of SCA. Validation experiments verified that the expression levels of DEGs in oncomine database are consistent with their expression level in GEO series. Hub genes were selected by PPI network, separately using 248 DEGs screened in the condition of |log_2_Fold Change| > 1.585 and 59 DEGs screened in the condition of |log_2_FoldChange| > 1.0. The intersection of the two hub genes sets helped us obtained 4 hub genes that highly interconnected with other nodes. Mutual exclusivity or co-occurrence analysis of 4 hub genes showed that there was a tendency towards co-occurrence between *KIF11* and *CDC20* or *TOP2A* in SCA (*p* < 0.05). Then, the co-expression profiles for *KIF11* obtained based on oncomine showed that *CDC20* co-expressed with *KIF11* in SCA, and they were also DEGs that screened out from SCA based on our previous results. The verified results from oncomine showed that the expression of DEGs in oncomine patient database were in accordance with data from GEO series. The survival curves show that the probability of SCA progression was found to be statistically significant with high *KIF11* and *CDC20* expression as compared to that of low *KIF11* and *CDC20* expression (*p* < 0.01). From all above results, we speculate that *KIF11* and *CDC20* play an important role in SCA. Though analyzed all GSE series compared SBOT and SCA tissues in GEO database, the prediction is more accurate and bias of individual studies can be overcome. Our study provides information for researchers to identify possible candidate genes and pathways which may be involved in SCA for further studies.

## Supplementary information

**Additional file 1: Figure S1.** Process of pooling 3 microarray gene expression datasets.

**Additional file 2: Figure S2.** Box figures of expression values of all genes before and after normalization. The results before and after normalization were showed by the top and bottom box-plots describe the expression values of 89 samples from GSE36668, GSE27651 and GSE12471 datasets. The yellow column represents the samples from GSE36668. The red column represents the samples from GSE27651. The blue column represents the samples from GSE12471. The 3 groups (yellow, red, blue) on the left were SBOT tissues and the 3 groups (red, blue, yellow) on the right were SCA tissues.

## Data Availability

The raw data supporting the conclusions of this manuscript will be made available by the authors, without undue reservation, to any qualified researcher.

## Abbreviations

SCA: Serous ovarian carcinomas
BOT: Borderline ovarian tumors
SBOT: Serous borderline ovarian tumors
log_2_FC: log_2_Fold Change/Logarithm of Fold Change
DEGs: Differentially Expressed Genes
PPI: Protein-protein interaction
GEO: Gene Expression Omnibus
*KIF11*: Kinesin Family Member 11
