# Supplementary figures and images for: Bioinformatics analysis of gene expression profile of serous ovarian carcinomas to screen key genes and pathways
Source: J Ovarian Res. 2020 Jul 21;13:82. doi: 10.1186/s13048-020-00680-1 (PMC7374965; doi:10.1186/s13048-020-00680-1)

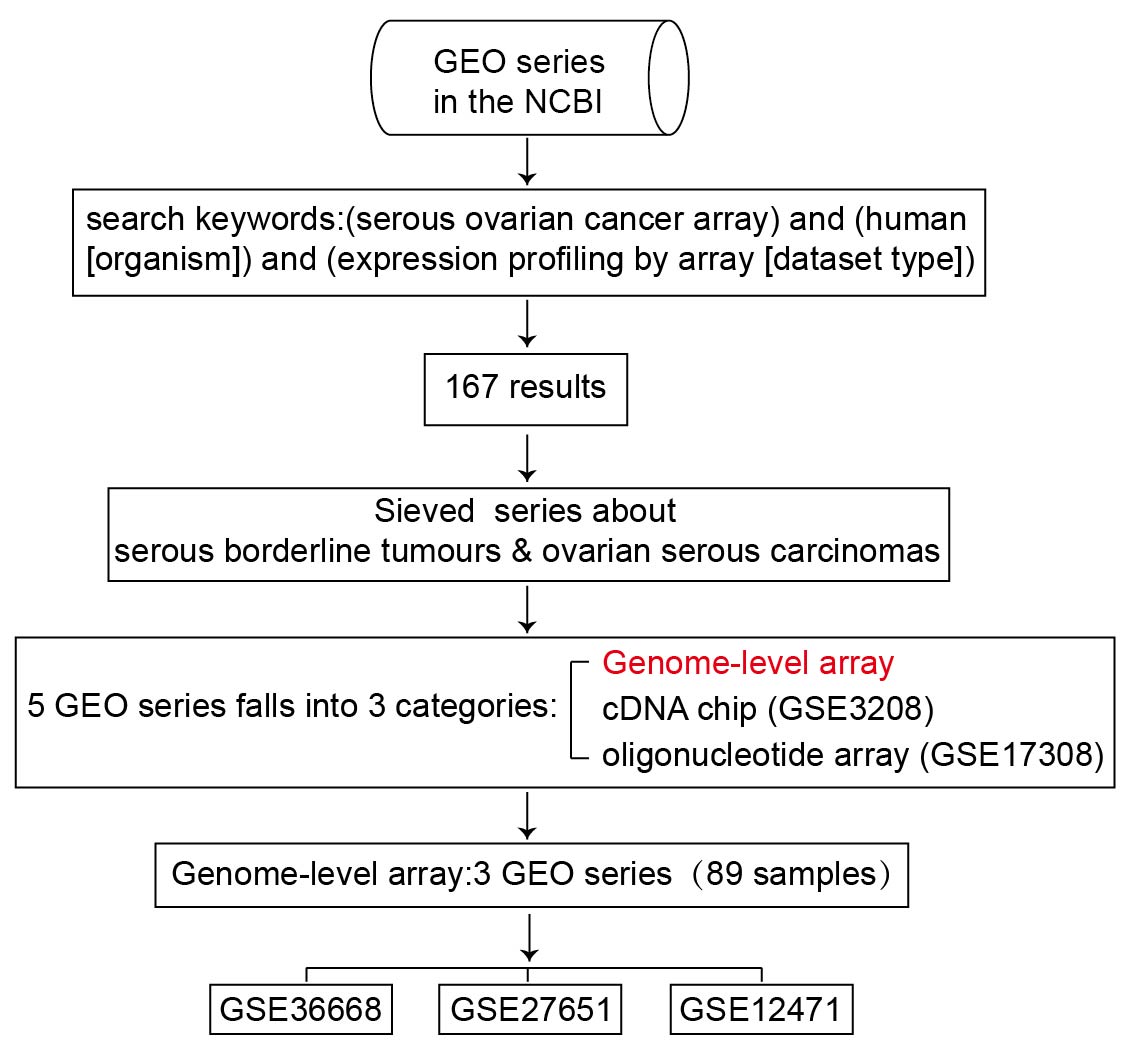

Supplement: Supplementary file 1 — Additional file 1: Figure S1. Process of pooling 3 microarray gene expression datasets. [file 13048_2020_680_MOESM1_ESM.jpg]

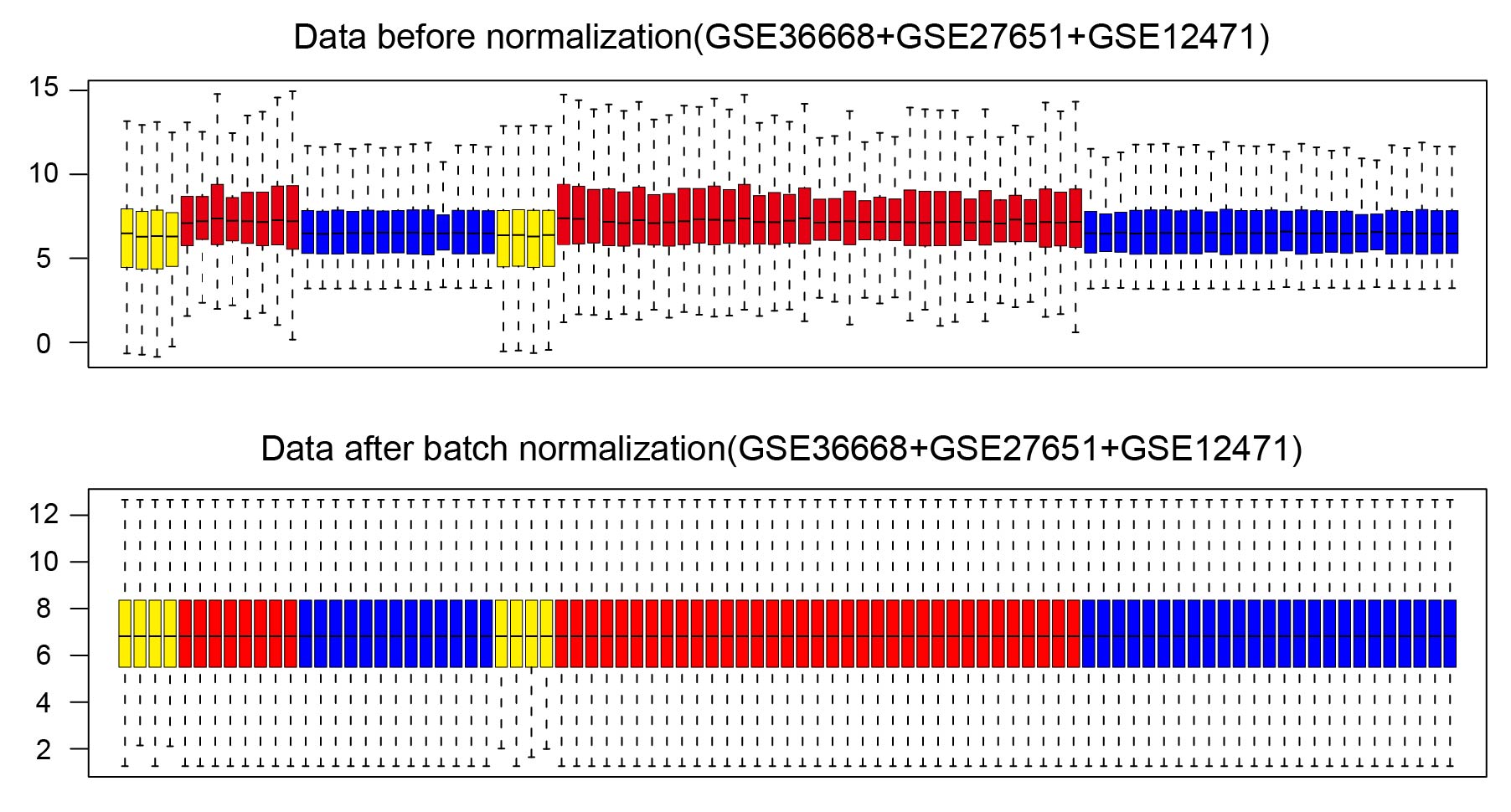

Supplement: Supplementary file 2 — Additional file 2: Figure S2. Box figures of expression values of all genes before and after normalization. The results before and after normalization were showed by the top and bottom box-plots describe the expression values of 89 samples from GSE36668, GSE27651 and GSE12471 datasets. The yellow column represents the samples from GSE36668. The red column represents the samples from GSE27651. The blue column represents the samples from GSE12471. The 3 groups (yellow, red, blue) on the left were SBOT tissues and the 3 groups (red, blue, yellow) on the right were SCA tissues. [file 13048_2020_680_MOESM2_ESM.jpg]
